# Evaluating clinical reasoning in first year DPT students using a script concordance test

**DOI:** 10.1186/s12909-024-05281-w

**Published:** 2024-03-22

**Authors:** Lindsey Kojich, Stephanie A. Miller, Katelyn Axman, Timothy Eacret, J. Atticus Koontz, Caroline Smith

**Affiliations:** University of Indianapolis, 1400 E. Hanna Ave, 46227 Indianapolis, IN USA

**Keywords:** Script concordance test, Physical therapy, Clinical reasoning, Assessment

## Abstract

**Background:**

A script concordance test (SCT) provides a series of clinical vignettes to assess clinical reasoning in uncertainty. Appraised throughout health education literature, SCTs are cognitive assessments of clinical reasoning, though their use in Doctor of Physical Therapy (DPT) entry-level education has not been investigated. The purpose of this study was to develop and explore the reliability and validity of a SCT for first year DPT students.

**Methods:**

The SCT was developed and implemented over four phases. During phases one and two, DPT program faculty consulted on course content from the first-year curriculum. Thirty clinical vignettes with three follow-up questions each were constructed. The SCT was pilot tested with five clinicians in phase three to assess question clarity. During phase four, the SCT was administered to students and a reference panel via Qualtrics. First year DPT students (*n* = 44) and reference panel physical therapists with at least two years of experience and advanced certification (*n* = 15) completed the SCT. Internal consistency was analyzed using Cronbach’s Alpha. Differences between student and reference panel percent-correct scores were analyzed with a t-test. Relationships between student SCT scores and academic records were explored with Spearman’s Rho.

**Results:**

The SCT had an internal consistency of 0.74. A significant difference in scores was found between the students [mean 58.5 (+/-5.31)] and reference panel [65.8 (+/-4.88), *p* < .01]. No significant correlations between student SCT scores and academic records were found.

**Conclusions:**

The developed SCT was reliable and demonstrated satisfactory internal consistency among test items. The SCT successfully differentiated between groups, with the reference panel demonstrating statistically significant higher percent-correct scores compared to students. SCTs may provide means to measure clinical reasoning in DPT students and lead to novel pedagogical approaches to enhance clinical reasoning.

## Background

Clinical reasoning in situations of ambiguity is a natural component of patient care. Doctor of physical therapy (DPT) students must develop the ability to adapt to the inevitable uncertainty that arises in the clinic, as demonstrated by successful practitioners [1]. While it has been well established that clinical reasoning is a vital component of practice, research on the assessment of clinical reasoning in physical therapy education is sparse [2, 3]. Additionally, many existing assessments can be time and resource intensive, limiting their use and implementation. A script concordance test (SCT), is one assessment tool not widely used in DPT education which could assist with the assessment of clinical reasoning in DPT students. The purpose of this study was to develop and analyze the reliability and validity of a SCT in first year DPT students.

**Fig. 1:**
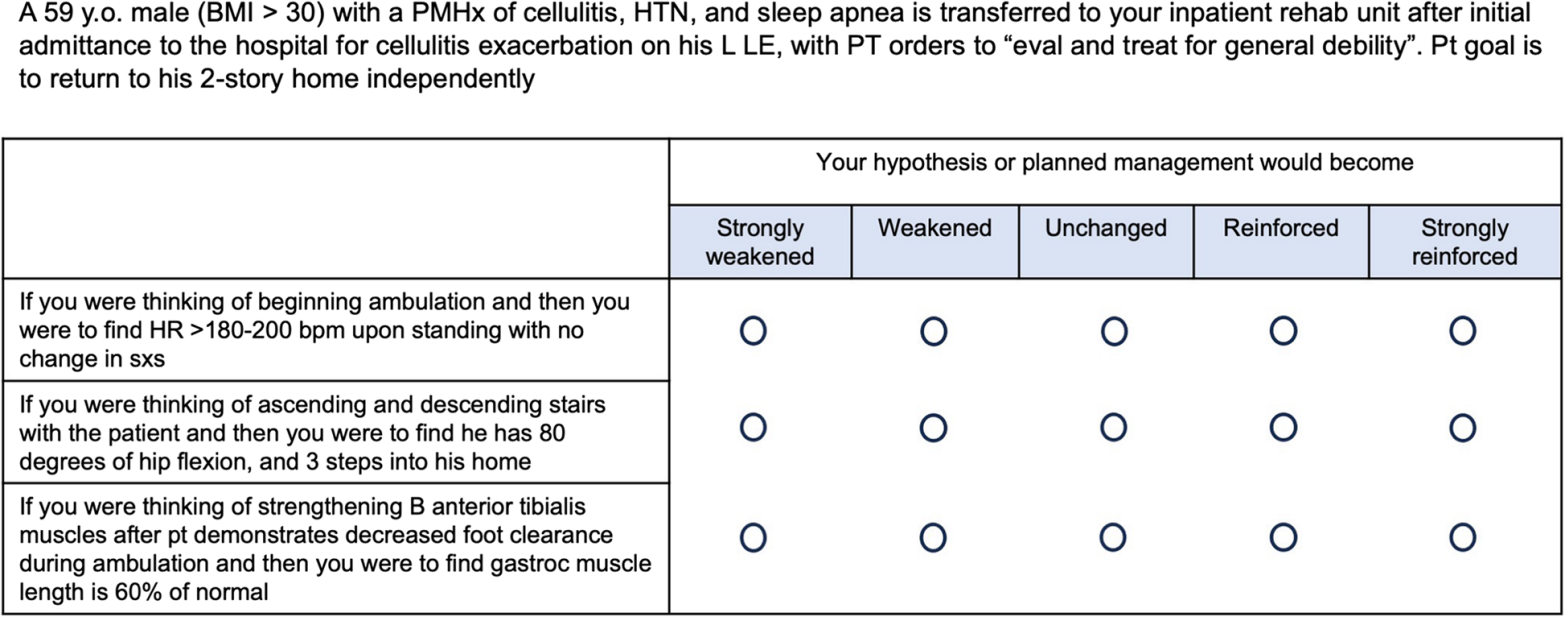
SCT Example

SCTs are easily administered, cognitive tests of clinical reasoning in ambiguity [4–6]. A SCT is constructed of a clinical vignette based on a real patient encounter, followed by a series of questions, as shown in Fig. 1. With each follow up question, the test taker is presented with new information for the case and then asked how the new data impacts their clinical judgment [5, 6]. The test taker answers on a 5-point Likert scale, with answer choices ranging from strongly reinforced to strongly weakened. Clinical vignettes and follow up questions are intentionally written so that there is no single correct answer, reflecting the variability that arises in practice [5, 6].

In addition to having a unique structure, SCTs are also scored differently than traditionally written assessments such as multiple-choice questions. SCTs are scored by comparing the answers of a target population, such as students or residents, to reference panels in the field [5, 6]. The most used scoring method for SCTs is aggregate scoring [4, 7, 8]. With aggregate scoring, all answers which correspond with the modal answer (the most frequently chosen answer) of the reference panel receive a score of 1. All other answers are given partial credit by dividing the number of reference panel members who chose that answer by the number of reference panel members who chose the modal answer [7, 8]. Table 1 displays an example of SCT scoring with 10 reference panel members. Five of the reference panel members chose strongly reinforced (+ 2), three chose reinforced (+ 1), and two chose unchanged (0). The student who chose strongly reinforced would receive 1 point for agreeing with the modal answer of the reference panel. Students who chose reinforced would receive partial credit of $^{3}/_{5}$  and students who chose unchanged would receive partial credit of $^{2}/_{5}$. The scoring of SCTs is unique in that it incorporates the natural variability of clinical practice in the structure and scoring of the test.

**Table 1:** Example of SCT scoring

	Strongly Weakened (-2)	Weakened (-1)	Unchanged (0)	Reinforced (+ 1)	Strongly reinforced (+ 2)
# of experts choosing	0	0	2	3	5
Credit for answer	0	0	2/5	3/5	1

The theoretical framework underlying SCTs is script theory [6, 9]. Script theory is derived from cognitive psychology and is based on the idea that health care providers organize, store, and access their knowledge through ‘illness scripts’ [5, 8–11]. These ‘scripts’ allow clinicians to use pattern recognition and previous clinical experience to draw from when making decisions during patient care [5, 8–11]. Scripts are formed throughout years of practice and assist the clinician in recognizing diagnoses, deciding treatment options, and making judgments on the many other tasks commonly involved in patient care [5, 9, 10]. Clinicians use illness scripts to reach decisions more efficiently and allow for improved decision making in novel situations. Research suggests that facilitating the development of illness scripts may enhance clinical reasoning in novice practitioners [9, 10]. Script concordance tests are based on script theory and their utilization may assist with pattern recognition and improved clinical reasoning and decision making in DPT students.

While the construction and validation of SCTs is expanding across several health professions including nursing, medicine, optometry, and dentistry, there is a paucity of research investigating their use in the field of physical therapy (PT). The majority of existing studies in PT focus primarily on SCTs as a means to evaluate clinical reasoning in licensed Physical therapists and specialties of practice, not in entry-level DPT education [12–15]. Cohen et al. explored the development and validity of a SCT for seating and mobility prescription in currently practicing physical therapists [12] while O’Brien et al. developed a SCT to assess growth in physical therapy residents’ clinical reasoning with assistive device prescription [13]. Both studies validated SCTs that were created for a specific content area in licensed and practicing physical therapists. Only one existing study has assessed clinical reasoning in physical therapy students. Otterman et al. investigated the use of SCTs to evaluate clinical reasoning in the field of stroke rehabilitation, comparing the scores of students to general practitioners and those specializing in neurology or geriatrics [15]. It is important to note that these students were undergraduate students pursuing a bachelor’s degree in PT. Thus, to the authors knowledge there have been no published studies investigating the use of SCTs to assess clinical reasoning in first-year DPT students.

The primary aim of this study was to develop and analyze the reliability and validity of a SCT for first year DPT students, compared to a reference panel of physical therapists. It was hypothesized that the newly developed SCT would identify differences in clinical reasoning between reference panel practitioners and first year DPT students, and that the SCT would have appropriate (high) internal consistency and demonstrate reliability of the vignettes and questions. The secondary aim was to investigate the relationship between student SCT scores and other programmatic assessments.

## Methods

### Study design

This study utilized a nonexperimental, single site, cross-sectional design to compare SCT scores between first-year DPT students and a reference panel of physical therapists. Correlations between first year DPT students SCT scores and programmatic assessments were explored. This study was approved by the Institutional Review Board of a midwestern University.

### Participants

Researchers recruited 44 first-year DPT student participants through convenience sampling from a midsized DPT program located in the Midwest from June 2, 2021 to June 16, 2021. Students were included in the study if they had completed two semesters of an entry-level DPT program and were in good academic standing at the time of the study. Forty-four of a possible 46 first-year students (95.65%) participated in the study resulting in a well-represented sample from the first-year class. Student participants were incentivized for participation with an electronic gift card. A sample size of at least 36 students was required to ensure adequate power for internal consistency and construct validity [16]. Reference panel members were recruited through convenience, purposive, and snowball sampling techniques from May 18, 2021 to July 7, 2021. Recruitment for reference panel members was conducted through university social media accounts and alumni association email. The reference panel consisted of licensed physical therapists from different geographical regions who practiced physical therapy in an outpatient clinic or hospital setting for at least 2–3 years and held advanced credentialing (American Board of Physical Therapy Specialties, Credentialed Clinical Instructor) in the profession. Reference panel members who had a specialty certification through the American Board of Physical Therapy Specialties must have practiced in the specialty for at least 2 years and reference panel members who had a Credentialed Clinical Instructor designation must have practiced for at least 3 years. Reference panel members were incentivized for participation with an electronic gift card. A reference panel size of 15 was determined based on literature citing that reference panels of 15–20 individuals are desirable for SCT reliability and validity [6, 17].

Demographics for the student and reference panel participants are presented in Table 2. The student sample (*n* = 44) is representative of the DPT student population with the majority of students being female (65.9%) and falling in the 18–24 age category (79.5%). Half of the student sample (50%) had prior experience in the healthcare field as a licensed professional, certified personal trainer, or physical therapy technician/ aide. The reference panel sample (*n* = 15) consisted of a wide range of years of experience (2–25 + years) and clinical specialty areas including orthopedics, cardiopulmonary, acute care, neurological, and pediatrics.

**Table 2:** Demographics of students and reference panel

Variable	Reference Panel	Students
	*N* = 15	*N* = 44
Age		
18–24	0 (0%)	35 (79.5%)
25–34	6 (40%)	8 (18.2%)
35–44	7 (46.7%)	1 (2.3%)
45–54	1 (6.7%)	0 (0%)
55–64	1 (6.7%)	0 (0%)
Sex		
Male	2 (13.3%)	15 (34.1%)
Female	13 (86.7%)	29 (65.9%)
Student prior experience		
Licensed professional		3 (6.8%)
CPT		7 (15.9%)
PT Tech/Aide		12 (27.3%)
None		22 (50%)
PT education		
DPT	14 (93.3%)	
BS	1 (6.7%)	
Years as a PT		
0–5	1 (6.7%)	
6–10	7 (46.7%)	
11–15	5 (33.3%)	
21–25	1 (6.7%)	
25+	1 (6.7%)	

Abbreviations: *CPT *Certified personal trainer, *PT Tech/ Aide *Physical therapy technician or physical therapy aide

### SCT development

**Fig. 2:**
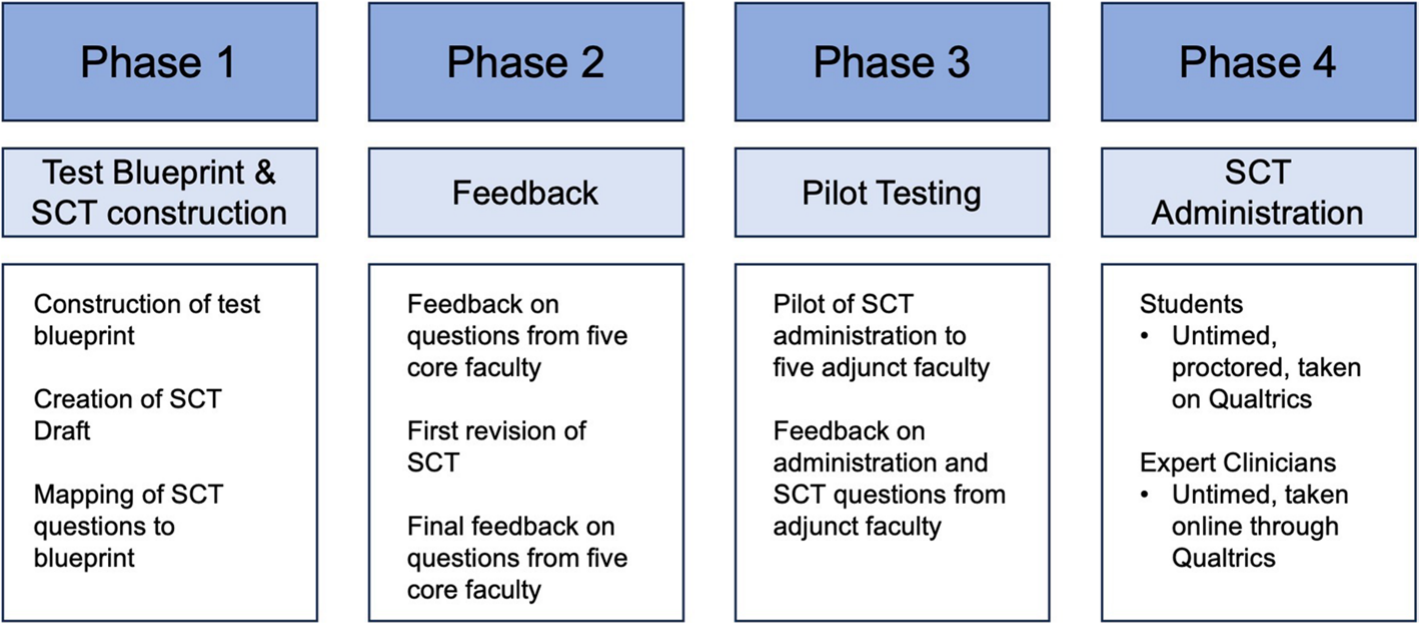
Phases of SCT development through administration

The SCT was developed and implemented over four phases: (1) Test Blueprint and SCT construction, (2) SCT feedback, (3) SCT pilot testing, and (4) SCT administration and data collection. These phases are summarized in Fig. 2.

### Phase 1: test blueprint and SCT construction

Prior to the creation of the SCT, a test blueprint was created to ensure the SCT was inclusive of content typical in the first year of a DPT curriculum. The test blueprint contained an outline of content areas and course objectives from the first year of a DPT curriculum at a midsized DPT program located in the Midwest. Course objectives from the curriculum were all mapped to the Commission on Accreditation in Physical Therapy Education accreditation standards, ensuring the SCT content was representative of the required elements of DPT education. The test blueprint was evaluated by five core DPT faculty members who were lead instructors of the first year of the curriculum. Using the Association for Medical Education in Europe (AMEE) guidelines [6], the preliminary draft of the SCT questions was designed from the test blueprint and all questions were mapped back to the blueprint to ensure appropriate representation of all subject matter. Questions were written to focus on physical therapy examination or intervention components of patient care.

### Phase 2: SCT feedback

Investigators shared the initial draft of the SCT with the same five core DPT faculty members contacted for the test blueprint. Faculty gave initial feedback about content validity, clarity, and relevance of the SCT questions to ensure the SCT captured appropriate content from the first year of the curriculum. Investigators then revised clinical scenarios and follow up questions in the SCT according to initial feedback from faculty. Poorly written questions were revised or discarded. The same feedback process was completed again with the second draft of the SCT before proceeding with pilot testing. The same core faculty reviewed the second draft of the SCT and provided feedback as necessary. The investigators finalized the SCT after the second round of feedback.

### Phase 3: SCT pilot testing

After phase two, investigators pilot tested the SCT with a convenience sample of five adjunct faculty members. To recruit faculty members for pilot test participation, researchers emailed all current DPT adjunct faculty who practice clinically through university email. The adjunct faculty taught at the same University and program conducting the study and met reference panel inclusion criteria. A three-minute pre-recorded instructional video was provided outlining SCT structure, the Likert scale, and how to take the SCT on the Qualtrics platform. The adjunct faculty members were asked to watch the video, then individually take the SCT. Answers for each question were reviewed by the research team. Any questions with unanimity of reference panels or extreme divergent responses were discarded or revised. Unanimity of responses suggest that the question is too straightforward, while divergent responses suggest the question is too ambiguous [6]. Suggestions and feedback about SCT instructions, use of Qualtrics platform, timing for completion, and content validity of the SCT were gathered from each adjunct faculty member to better inform administration of the optimized SCT.

### Phase 4: SCT administration and data collection

The optimized SCT was administered in the final phase of the study. All reference panel physical therapists who were deemed eligible for the study and indicated an interest to participate were emailed a link to the Qualtrics survey to participate in the study virtually. None of the reference panel physical therapists participated in the first three phases of SCT development. All students who were deemed eligible for the study and who indicated an interest were provided with details of the testing date, time, and location. The SCT was administered to all student participants in person, in a proctored room, in order to ensure test integrity and that all students completed the SCT independently. After indicating consent to participate, all participants completed a demographic survey, watched a three-minute pre-recorded instructional video outlining how to take the SCT on the Qualtrics platform, followed by taking the SCT. Participants were not timed and could take as much time as needed to complete the SCT.

Academic records were collected of student participants. This data included cumulative grade point average (GPA) in the DPT program after two semesters, cumulative and prerequisite GPA upon admission to the DPT program, and individual course grades. Additionally, final scores on an objective structured clinical examination (OSCE) and benchmark exam given at the end of the first year were collected. The OSCE is a summative assessment involving examination, evaluation and intervention on a simulated patient. It is evaluated on a grading rubric by a faculty member. The benchmark exam is a cumulative, written, multiple-choice exam designed to test knowledge of the first year of the curriculum and simulate the national physical therapy examination.

### Data analysis

Descriptive statistics were conducted on both groups and nominal data were presented as frequencies and percentages; continuous data were presented as means and standard deviations. Investigators exported SCT data from Qualtrics and utilized the aggregate scoring method to score the SCT through Excel. The aggregate scoring method is the most common scoring method referenced in health professions literature [4, 5]. Investigators calculated modal answers by identifying the most frequently chosen answer for each question from the reference panel. Modal answers received a score of + 1. Non-modal answers were calculated by taking the number of answers for the non-modal answer from the reference panel divided by the number of individuals choosing the modal answer from the reference panel. Thus, non-modal answers received a score between + 0 to + 0.99. After scoring, test optimization occurred to ensure quality of the SCT items according to AMEE Guidelines [6]. Items with bimodal responses, where reference panels’ answers were split between the two extremes (-2 and + 2) were discarded due to extreme discordance to better optimize the test [18]. Additionally, items with unanimity of the reference panel were also discarded as this response pattern indicates the question was poorly written and too straight forward [6]. Student SCT responses were scored based on the modal and non-modal answers from the reference panel and then compared to reference panel responses. Normality of data was tested using the Shapiro-Wilk test and Alpha was set a priori at *p* ≤ .5. Cronbach’s alpha was computed to evaluate internal consistency of SCT items. A Cronbach’s alpha between 0.7 and 0.9 was desired, higher than 0.9 was interpreted as redundant constructs, and lower than 0.7 was interpreted as measuring different constructs [8]. Differences between reference panel and student percent-correct scores on the SCT were examined with a t-test. SCT and OSCE scores were normally distributed and all other assessments were not normally distributed. Relationships between student SCT scores and existing academic records were explored with Pearson’s correlations for parametric data and Spearman’s Rho for nonparametric data. All analyses were conducted using IBM Statistical Package for the Social Sciences (SPSS) version 25.0 (SPSS, Inc., Chicago, IL).

## Results

**Fig. 3:**
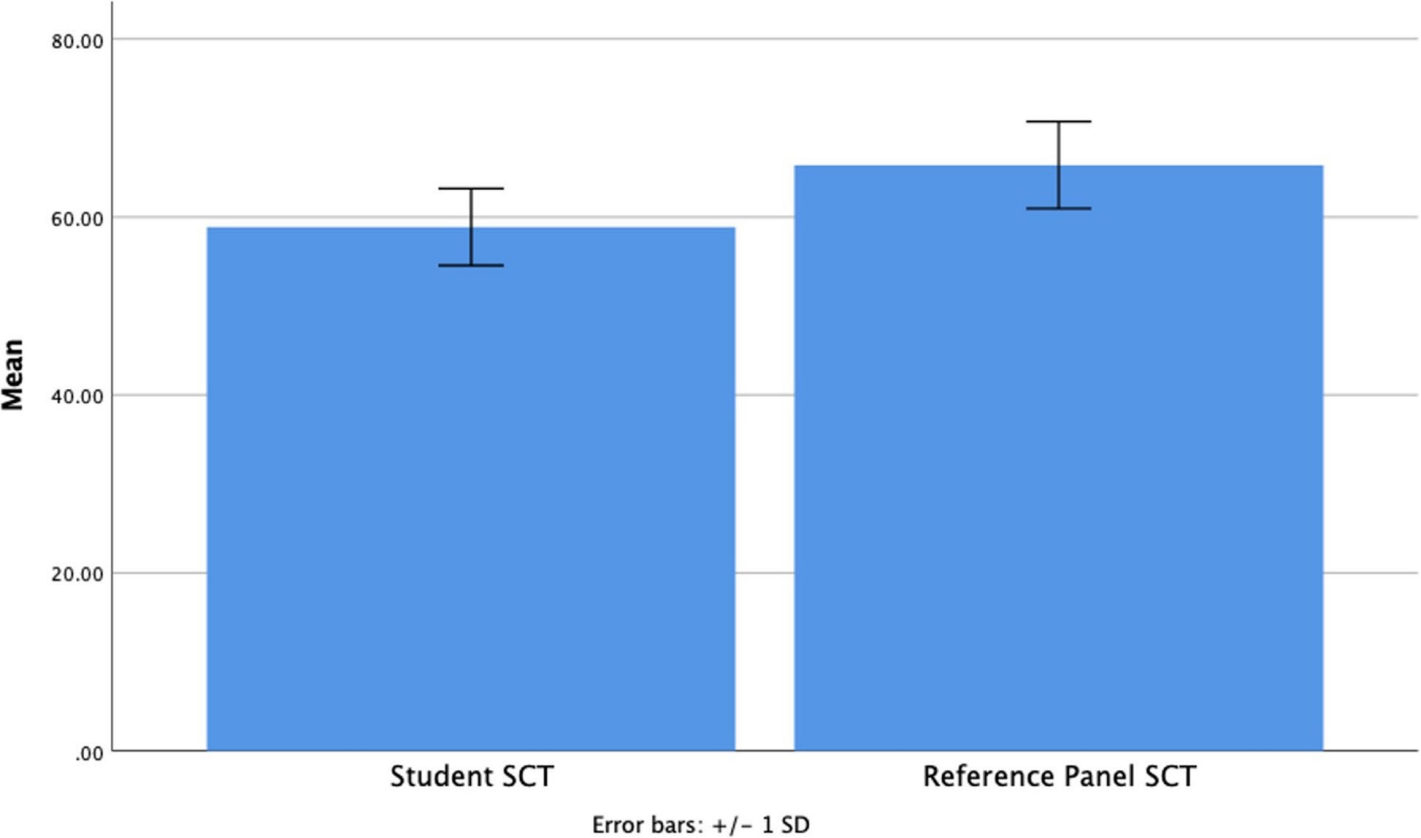
Bar chart of student and reference panel SCT scores

Thirteen items were eliminated for test optimization, resulting in a 77 item SCT. The final SCT had a Cronbach’s alpha of 0.74, indicating satisfactory consistency among test items. A significant difference in percent-correct scores was found between students and the reference panel [students mean (SD) 58.5 (5.31) and reference panel mean (SD) 65.8 (4.88), *p* < .01], as shown in Fig. 3. A correlation matrix of student SCT scores and academic records is presented in Table 3. No statistically significant correlations between student SCT percent-correct scores and academic records were found.

**Table 3:** Correlation matrix of SCT percent-correct scores and academic records

	SCT	OSCE	Bench-mark	GPA	Clin Med 1	Clin Med 2	Clin Skills 1	Clin Skills 2	PtCare Skills	Gross Anatomy
SCT	1.00^+^									
OSCE	0.024^+^	1.00^+^								
Benchmark	0.133	0.182	1.00							
GPA	− 0.052	0.484^**^	0.250	1.00						
ClinMed1	0.03	0.256	0.181	0.821^**^	1.00					
ClinMed2	0.067	0.280	0.058	0.782^**^	0.806^**^	1.00				
ClinSkills1	− 0.079	0.548^**^	0.192	0.830^**^	0.568^**^	0.649^**^	1.00			
ClinSkills2	− 0.019	0.487^**^	0.281	0.849^**^	0.677^**^	0.689^**^	.786^**^	1.00		
PtCareSkills	− 0.093	0.255	0.018	0.350^*^	0.279	0.170	0.279	0.305^*^	1.00	
Gross Anatomy	− 0.130	0.389^**^	0.208	0.841^**^	0.588^**^	0.470^**^	0.701^**^	0.632^**^	0.191	1.00

Abbreviation: SCT, Script Concordance Test; OSCE, Objective structured clinical examination; GPA, First year cumulative GPA

^a^ Double ** indicates correlation is significant at the 0.01 level (2-tailed)

^b^ Single * indicates correlation is significant at the 0.05 level (2-tailed)

^c^ Single ^+^ indicates Pearson’s correlation was conducted

## Discussion

This is the first study examining the development of a SCT for clinical reasoning in first-year DPT students. The newly developed SCT was reliable with satisfactory internal consistency. Cronbach’s alpha of the SCT (0.74), was within the desired range of 0.7–0.9, indicating good consistency among test items. This finding is consistent with the literature, as SCTs in the fields of medicine, nursing, and optometry found Cronbach’s alpha values ranging from 0.65 to 0.90 [19–26].

A significant difference between percent-correct scores of first year DPT students and reference panel members was found (*p* < .01), confirming that the first-year DPT student SCT was capable of distinguishing between individuals with different levels of clinical experience. Several prior studies in medicine have found significant differences between physicians, residents, and students of differing experience levels using individually developed SCTs [21, 23, 25]. Omega et al. performed a cross-sectional study validating a SCT on emergency airway management [27]. Researchers found that the SCT was able to discriminate between junior, intermediate, and senior anesthesiology residents. A similar study performed by Wan et al. found that a SCT covering multidisciplinary medical topics was discriminatory between a reference panel and students, and that fourth-year medical students’ scores were higher than third year students’ scores [28].

Perhaps the most interesting finding of this study was the lack of relationship between DPT student SCT scores and other curricular assessments. No correlation was found between students’ SCT scores and their cumulative and prerequisite GPAs or individual course grades in the first year of the curriculum. Additionally, there was no correlation between students’ SCT scores and their OSCE or benchmark exam scores. These findings may suggest that the SCT is measuring a skill, clinical reasoning in uncertain situations, that is not currently captured by the knowledge-based examinations traditionally used to evaluate DPT students throughout the first-year curriculum. Most of the assessments performed in the first year mimic the multiple-choice question format of the National Physical Therapy Examination (NPTE) which does not have a specific subsection dedicated to the examination of clinical reasoning. Lack of relationships between SCT scores and OSCE scores may be due to SCTs evaluating cognitive aspects of clinical reasoning in uncertainty while OSCEs evaluate not only cognitive but also psychomotor and affective skills.

Several researchers in medicine have investigated possible correlations between SCTs and other programmatic assessments. Humbert et al. developed and validated an SCT in emergency medicine (SCT-EM) and found a significant correlation between fourth year medical student SCT-EM scores and performance on two summative medical exams, the American Board of Emergency Medicine (ABEM) in-training exam, and the Step 2 Clinical Knowledge (CK) Exam [29]. Interestingly, eight years later, Steinberg et al. administered the same SCT-EM to emergency medicine residents and attendings and reported no significant correlation between residents’ SCT-EM scores and emergency medicine milestone scores set by the Accreditation Council for Graduate Medical Education [25]. These differences may be due to the timing in students’ level of education, or the nature of the assessments, as the ABEM in-training and Step 2 CK exams are comprehensive written exams while the emergency medicine milestone scores are clinical performance based. A literature review from Lubarsky et al. also found weak correlations between SCTs and knowledge-based examinations in studies from the medical field, however, more studies are needed to explore these findings [8].

SCTs are unique in that they accept the variation in patient care that may exist amongst clinicians. Whereas traditional written assessments are more knowledge based, and focus on one correct answer, SCTs ask test takers to make decisions in ambiguous situations with scores that are reflective of concordance with reference panels [4, 8]. Additionally, SCTs are intentionally designed to assess higher level thinking and to incorporate uncertainty [30, 31]. Knowledge based exams may incorporate more lower-level knowledge skills such as recalling or understanding facts, and typically do not intentionally include ambiguity. This may account for the lack of correlation between SCT scores and all other academic scores/grades. Findings from a mixed methods study by Ottolini et al. supports this hypothesis, as pediatric hospitalists taking both a SCT and multiple-choice question exam reported the SCT questions elicited higher order thinking and reasoning compared to multiple-choice question exams [31]. Given that clinical practice is multidimensional, and every assessment has strengths and weaknesses, utilizing multiple types of assessment instruments throughout curricula may assist in gaining a better understanding of a student’s overall performance [1]. The addition of SCTs as an assessment method in DPT education may provide valuable insight into students’ reasoning in ambiguity.

It is important to note that the construction and scoring of SCTs is variable among the literature. In this study, the SCT was developed according to the guidelines for construction detailed in Fournier et al. [5]. Additional types of SCTs have been created involving an alternate follow up question creation or qualitative components. Cooke et al. developed what they termed an “evolving SCT” for pediatricians, whereby the clinical decisions become increasingly clear with each new piece of information within the follow-up questions in each clinical vignette [19]. Recently, there have been several other studies which have implemented a “think aloud” approach in their SCTs, where a short-answer section following each question allows students and clinicians to provide reasoning for their answer [32, 33]. Think aloud SCTs have also been investigated as a formative assessment of clinical reasoning. These types of SCTs deserve further investigation as to their utility in assessing clinical reasoning, and as a pedagogical tool to improve clinical reasoning [34, 35].

### Study limitations

There were several limitations to this study. Data were collected using a single DPT cohort at one institution, limiting external validity. Furthermore, the SCT was developed from the content of one DPT curriculum. The authors believe this material is standard for the majority of DPT programs, but there are likely a number of institutions with differing curricular structure for whom this SCT would not be applicable to first year students. While there are a variety of methods that have been postulated to impact clinical reasoning in DPT students, there is still a large gap in the literature on facilitating and assessing clinical reasoning. In this study, no correlation between student SCT scores and other curricular evaluation methods was found, indicating clinical reasoning in uncertain situations may not be effectively assessed in DPT education. Additional study is warranted investigating the utility and validity of SCTs in DPT education as either an assessment or pedagogical tool. Future directions of this work include administering the SCT to multiple cohorts overtime to determine whether a significant change in clinical reasoning skills will be observed. Also, expanding the sample of first-year students from other DPT programs may provide greater strength and generalizability of the findings.

## Conclusion

The newly developed SCT is reliable with satisfactory internal consistency among test items. The SCT successfully differentiated between groups, with reference panels demonstrating significantly higher percent-correct scores compared to students. Lack of significant relationships between SCT scores and academic records may indicate that the SCT measures the construct of clinical reasoning, not typically captured in traditional examinations used in DPT education. Further exploration of the utility of SCTs to assess clinical reasoning is needed in DPT students. Accordingly, improved methods of assessment could lead to novel pedagogical approaches to enhance clinical reasoning.

## Data Availability

The datasets used and analyzed during the current study are available from the corresponding author on reasonable request.

## Abbreviations

DPT: Doctor of Physical Therapy
SCT: Script concordance test
AMEE: Association for Medical Education in Europe
OSCE: Objective structured clinical examination
GPA: Grade point average
MCQ: Multiple choice question
SCT-EM: Script concordance test – emergency medicine
ABEM: American Board of Emergency Medicine
CK: Clinical knowledge
PT: Physical therapy
